# Supplementary material for: Evaluation of School Children Nutritional Status in Ecuador Using Nutrimetry: A Proposal of an Education Protocol to Address the Determinants of Malnutrition
Source: Nutrients. 2022 Sep 6;14(18):3686. doi: 10.3390/nu14183686 (PMC9502477; doi:10.3390/nu14183686)
Supplement: Supplementary file 1 [file nutrients-14-03686-s001.zip › nutrients-1873439-supplementary.pdf]

Table S1. Intervention planning matrix with intervention objectives, indicators, sources of verification and activities focused primarily on the elementary school setting.

**Intervention objectives**	**Indicators**	**Verification sources**	**Activities**
Involve the municipality's health center and town hall, a university education center, parents' associations, teachers and school authorities to guarantee autonomy and sustainability.	At least two meetings per year with and among the managers/representatives of all sectors that are to be involved.	Attendance record according to the established schedule.	- Generate written agreements with the local authorities stating that they will collaborate in order to guarantee their participation.<br>- Take measures to improve sanitation in the area by the municipality and ensure equitable access to drinking water.<br>- Ensure health assistance by the health center when necessary, in addition to its involvement in the promotion of health in the area.<br>- Include incentives for collaborating with the community in one or more subjects (extra points or credits) in the university center to motivate student participation, as well as offering final degree or master's degree projects related to the topic.
Train university students, teachers and school authorities for all anthropometric data collection activities, educational activities, monitoring, evaluation and problem detection.	At least 70% of students, teachers and authorities will be properly qualified for all collection, intervention, monitoring, evaluation and problem detection activities.	- Record of attendance to scheduled training sessions.<br><br>- Difference in knowledge between the two tests performed (one prior to the start of the training and the other at the end).	- Provide informative talks and interactive workshops adapted to each population according to their level of knowledge.<br>- Train the trainer of trainers by establishing a circle to ensure autonomy and sustainability.

Improve knowledge and attitudes regarding healthy lifestyle habits (food, hygiene and health, and physical exercise) of schoolchildren, parents, teachers and school authorities.	Knowledge of healthy lifestyle habits will improve by at least 50% in the first year of the intervention.	- Record of attendance to programmed activities.<br><br>- Difference in knowledge between the three tests performed (one prior to the start of the intervention training, the second 6 months later, and the third 1 year after the first).	- Provide didactic and interactive talks and workshops adapted to each population group according to their age and educational level.<br>- Include classes on healthy habits in at least one subject of the primary education curriculum.<br>- Ensure that, in physical education, all students interact and that different activities and sports are included to ensure that they are motivated and that their needs adapted to.<br>- Place advertising posters in strategic places within the schools.<br>- Hand out diptychs and triptychs with relevant information.<br>- Supervise the food served at school cafeterias.<br>- Form school cooking groups, "Master chef", where students are taught how to prepare healthy meals with seasonal and local foods.<br>- Mark walking trails within the schools so that students can walk them during recesses.<br>- Invite a well-known athlete to the schools to share their experience.<br>- Create ecological vegetable gardens in schools.<br>- "Sports week": allocate a few curricular hours during the week to promote sports competitions among students, teachers, authorities and parents.<br>-"Fair: Healthy Family Day": allocate one annual day where students, teachers, authorities and legal representatives prepare and share together, exhibitions and workshops are organized, and healthy activities are learned.

Reduce school malnutrition figures.	Malnutrition will decrease by at least 10% in the first year of the intervention.	Difference in prevalence percentages determined with Nutrimetry between the first intake (pre-intervention) and second intake of anthropometric values (post-intervention).	- Provide deworming/supplementation services for schoolchildren in need at the nearby health center.<br>- Refer cases to specialized health personnel (in nearby medical center) that need specific interventions, and continue to follow them up.<br>- Provide deworming services for teachers, authorities and parents who are in direct contact with the children if necessary.<br>- Take at least two anthropometric measurements to verify the improvement of nutritional status per year at the population level and at the clinical level so that the improvement in the follow-up will be greater.
